# Assessing effects of temperature driven by sowing dates on the grain filling and kernel weight of corn in the North China Plain

**DOI:** 10.3389/fpls.2025.1539207

**Published:** 2025-09-02

**Authors:** Panpan An, Kui Liu, Moubiao Zhang, Shunli Zhou, Xucun Jia, Xiuling Wang, Pengfei Dong, Yali Zhao, Qun Wang, Chaohai Li, Tianxue Liu

**Affiliations:** ^1^ Collaborative Innovation Center of Henan Grain Crops/Co-construction State Key Laboratory of Wheat and Maize Crop Science, Agronomy College, Henan Agricultural University, Zhengzhou, Henan, China; ^2^ Swift Current Research and Development Centre, Swift Current, SK, Canada; ^3^ College of Agronomy and Biotechnology, China Agriculture University, Beijing, China

**Keywords:** corn, sowing dates, kernel weight, temperature difference, adaption

## Abstract

**Introduction:**

The grain filling process is strongly influenced by weather during the reproductive growth stage, but grain filling and yield of corn under various weather conditions resulting from different sowing dates remain uncertain.

**Methods:**

A two-year study was conducted at Henan Agricultural Research Farm in 2015 and 2016 to investigate the grain filling dynamics of corn under different temperatures that were modified through four distinct sowing dates: spring sowing in late April, early summer sowing in late May, summer sowing in mid-June, and autumn sowing in early July.

**Results:**

The findings indicated that the elevated temperatures in 2016 led to an 8.7% reduction in yield and a 7.4% decrease in 1000-kernel weight compared to the normal temperatures in 2015, attributed to a shortened grain filling period resulting from the higher temperature (31.6°C). Significant differences in yield were observed across the four sowing dates. Findings from this study also highlighted that growing degree-days had a weak direct, but a strong indirect positive effect on thousand kernel weight through the grain filling duration. The average daily temperature had a weak direct negative, but a strong indirect positive effect on kernel weight through mean grain filling rate.

**Discussion:**

With the global climate changing, the intensity and frequency of high temperatures are expected to increase. To reduce corn yield loss due to high temperature in North China Plain advancing sowing dates from mid-June to late May is recommended. It is concluded that early sowing could effectively prolong the active grain filling duration and offset the decline in grain filling rate caused by high temperatures. The findings demonstrated advancing sowing dates improved the grain filling and yield of summer corn, thereby mitigating high temperature stress under global warming.

## 1 Introduction

A global climatic warming trend has been documented during the last several decades, and this trend is projected to accelerate in the future, increasing the risk of food insecurity globally (Zhao et al., 2017; Wing et al., 2021; Farooq et al., 2022; Wheeler and von Braun, 2013). Global warming, reaching 1.5°C above pre-industrial levels during 2021-2040, is expected to cause unavoidable increases in multiple climate hazards and multiple risks to ecosystems and cause variable precipitation patterns across most land areas (IPCC, 2022). Therefore, agricultural systems need to adapt to the anticipated warmer and more extreme climates to maintain or increase productivity (Zhu et al., 2018).

Corn is the world’s largest cereal crop in terms of total production and plays a critical role in ensuring food security (Wheeler and von Braun, 2013). The North China Plain (NCP) is one of China’s most important grain production areas (Zhang et al., 2013), which has an annual corn-sown acreage of approximately 15 million hectares and accounts for roughly 29.0% of national corn production (National Bureau of Statistics of the People’s Republic of China, 2014, https://data.stats.gov.cn/). Crop production in the NCP is sensitive to changes in both temperature and management practices (Liu et al., 2010) and has been impacted by global warming, which has led to more frequent and intensified of heat stress during grain filling seasons increasing in recent decades, causing substantial yield losses (Wang et al., 2021). Corn grain yield is primarily determined by kernel number (Bonelli et al., 2016; Tsimba et al., 2013), but variations in kernel weight can affect corn yield (Bonelli et al., 2016; Zhou et al., 2017; An et al., 2018; Borrás and Gambín, 2010). Kernel weight is influenced by grain filling parameters, such as the average filling rate, effective grain filling duration, and middle grain filling stage (Jia et al., 2018; Jiang et al., 2016; Mirosavljević et al., 2018).

The dynamics of kernel mass accumulation may be significantly affected by weather conditions such as high temperatures, solar radiation, and precipitation (Edreira et al., 2014; Gouache et al., 2012). High temperatures accelerate corn grain filling rate but shorten the growth duration; however, the increase in grain filling rate does not compensate for the reduced duration, thus resulting in lower grain yield (Farooq et al., 2011; Kim et al., 2011; Mayer et al., 2014). High temperature was the primary cause of aborted kernels, shortened grain filling duration, leading to a reduction of kernel weight (Barnabás et al., 2008). When corn is exposed to daily maximum temperatures≥35°C, the grain filling period was shortened by 6–17 days compared to corn exposed to a 26°C temperature (Tao et al., 2016b). A decrease in solar radiation during the grain filling period caused a reduction in corn grain yield (Hatfield, 2014), although some studies pointed out that solar radiation is not a limiting factor for the low production of summer corn (Hu et al., 2019). Precipitation during corn growing seasons showed a decreasing trend in the northeast China from 1981 to 2010 (Liu et al., 2012). During the year from 1954 to 2014, temperature increased significantly during both vegetative and reproductive growing periods of corn in the NCP with a slight decline in precipitation (Huang et al., 2018). With the widespread adoption of modernized agricultural machinery in recent years, corn sowing date is typically delayed by 10 days in the NCP. Such delay is due to the conversion from a winter wheat-summer corn relay cropping system to a rotation where corn is sown after wheat harvest. Despite the importance of corn production in the NCP, few studies have examined the influence of cropping systems and weather variability on the dynamics of kernel weight and grain filling, which contributes to developing strategies to adapt to climate change in corn production. To address these issues, we conducted a two-year field experiment to investigate the impact of temperature difference created by different sowing dates on grain filling process and yield of summer corn. Understanding the response of kernel weight to the temperature variance resulting from different sowing dates and the mechanisms governing the grain filling process is essential for developing strategies to adapt to climate change in corn production. The study aims to understand the response of kernel weight to weather variance resulting from different sowing dates, providing essential insights for developing effective corn production strategies to mitigate anticipated high temperature stress under projected global warming.

## 2 Materials and methods

### 2.1 Site description

**Figure 1:**
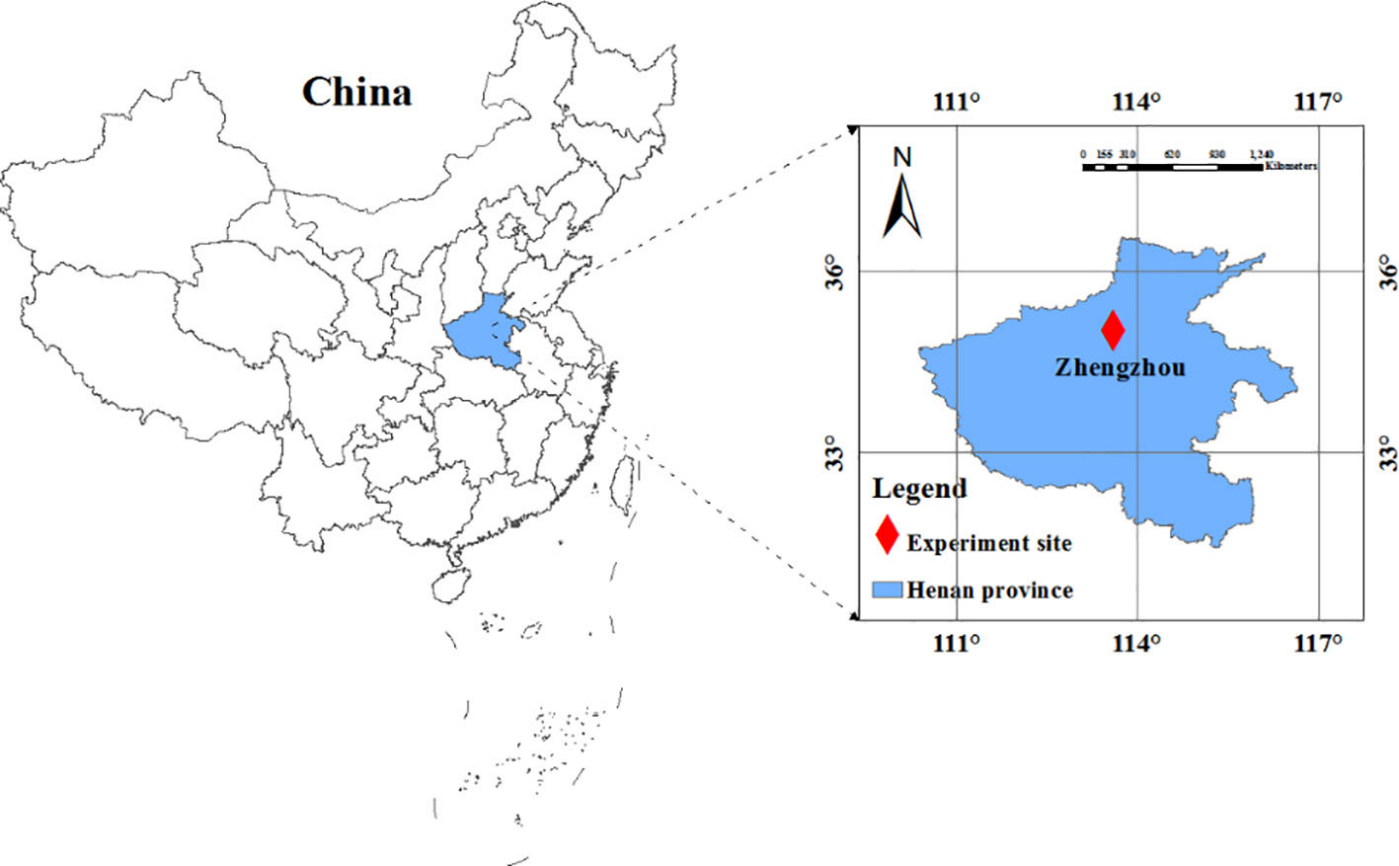
The location of experimental site.

A two-year study (2015 and 2016) was conducted in 2015 and 2016 at Henan Agricultural Research Farm located in Zhengzhou, Henan Province, China, as shown in **Figure 1**. The study site has a long-term average annual precipitation of 609.5 mm, a mean temperature of 14.7°C, and a frost-free period of 216 d. The baseline soil fertility in the 0–20 cm soil layer was as follows: soil organic matter 9.18 g kg^-1^, total N 0.98 g kg^-1^, alkaline-extractable N 63.5 mg kg^-1^, Olsen-extractable P 22.8 mg kg^-1^, and Olsen-extractable K 133.8 mg kg^-1^.

### 2.2 Experimental design

This study includes four sowing date treatments: spring sowing in late April (SSA), early summer sowing in late May (SSM), summer sowing in mid-June (SSJ), and autumn sowing in early July (ASJ). Sowing occurred from late April to early July to capture the earliest and latest dates suitable for sowing corn in the study region. Each plot was 6 m long and 3.6 m wide and consisted of 6 rows. A randomized complete block design with three replications was used for this study. The weather variables for each sowing date treatment at the experimental site are presented in **Table 1**. A widely grown corn cultivar, Zhengdan 958 (ZD958) was chosen for this study because of its high yield, resistance to multiple stressors, and extensive adaptability. Before sowing, plots were finely prepared with a disk plow. Corn was planted at a row spacing of 0.6 m and plant spacing of 24.7 cm. To achieve uniform plant density, all plots were thinned at the V4 stage to achieve population densities of 67,500 plants ha^-1^, which was recommended by the local corn grower guide to maximize grain yield under unstressed conditions.

**Table 1:** Weather conditions during the corn reproductive growth stage from June 20th to October 20th across different sowing dates for the 2015 and 2016 growing seasons and the 30-year (1987-2016) average.

Date of reproductive stage	30-year average				2015				2016			
	DSD	ADT	EP	GDD	DSD	ADT	EP	GDD	DSD	ADT	EP	GDD
June 20^th^-July 20^th^	176.1	27.2	140.3	541.2	140.4	26.1	143.6	506.6	186.8	27.8	256.3	559.5
July 21^th^-Aug.20^th^	172.9	27.1	126.5	540.9	153.6	28.4	91.1	574.8	186.4	29.4	71.3	613.4
Aug.21^th^-Sep.20^th^	162.7	23.2	95.8	423.3	177.4	23.3	111.5	424.2	184.7	26.0	60.3	505.0
Sep.21^th^-Oct.20^th^	154.1	18.1	44.0	263.9	162.7	20.4	34.9	322.7	129.1	20.0	52.7	309.8
Total	665.8		406.6	1769.3	634.1		381.1	1828.2	687.0		440.6	1987.7
Mean		24.0				24.5				25.9		

DSD, daily sunshine duration; ADT, average daily temperature; EP, effective precipitation; GDD, growing degree-days.

The fertilizer application rates were determined based on soil test recommendations. Basal fertilizers were applied at a rate of 90 kg N ha^-1^, 120 kg P_2_O_5_ ha^-1^, and 50 kg K_2_O ha^-1^. Furthermore, additional N fertilizer (135 kg N ha^-1^) was applied at the 12th leaf stage (V12). The experimental field was first irrigated using sprinkler systems with groundwater as the water source. The sprinklers systems were placed in the middle of each row. The sprinklers spacing was 3 cm, and the flow rate was 0.32 L h^−1^ at an operating pressure of 0.1 MPa (Tianye Inc., China). Each plot was connected to a high precision water meter (LXS-40F, Ningbo, China) and control valve. The irrigation level was determined based on the local irrigation quota. The irrigation generally occurred two or three times depending on rainfall. The maize was irrigated immediately after sowing and was irrigated again at the V12 stage, following a split nitrogen application. The total amount of irrigation water applied ranged from 50 to 75 mm based on crop requirement. The local best management practices were followed for achieving high yield. All experimental fields were well-managed, and no water or drought stress or pest damage was observed during the growing season.2.3 Weather data

**Figure 2:**
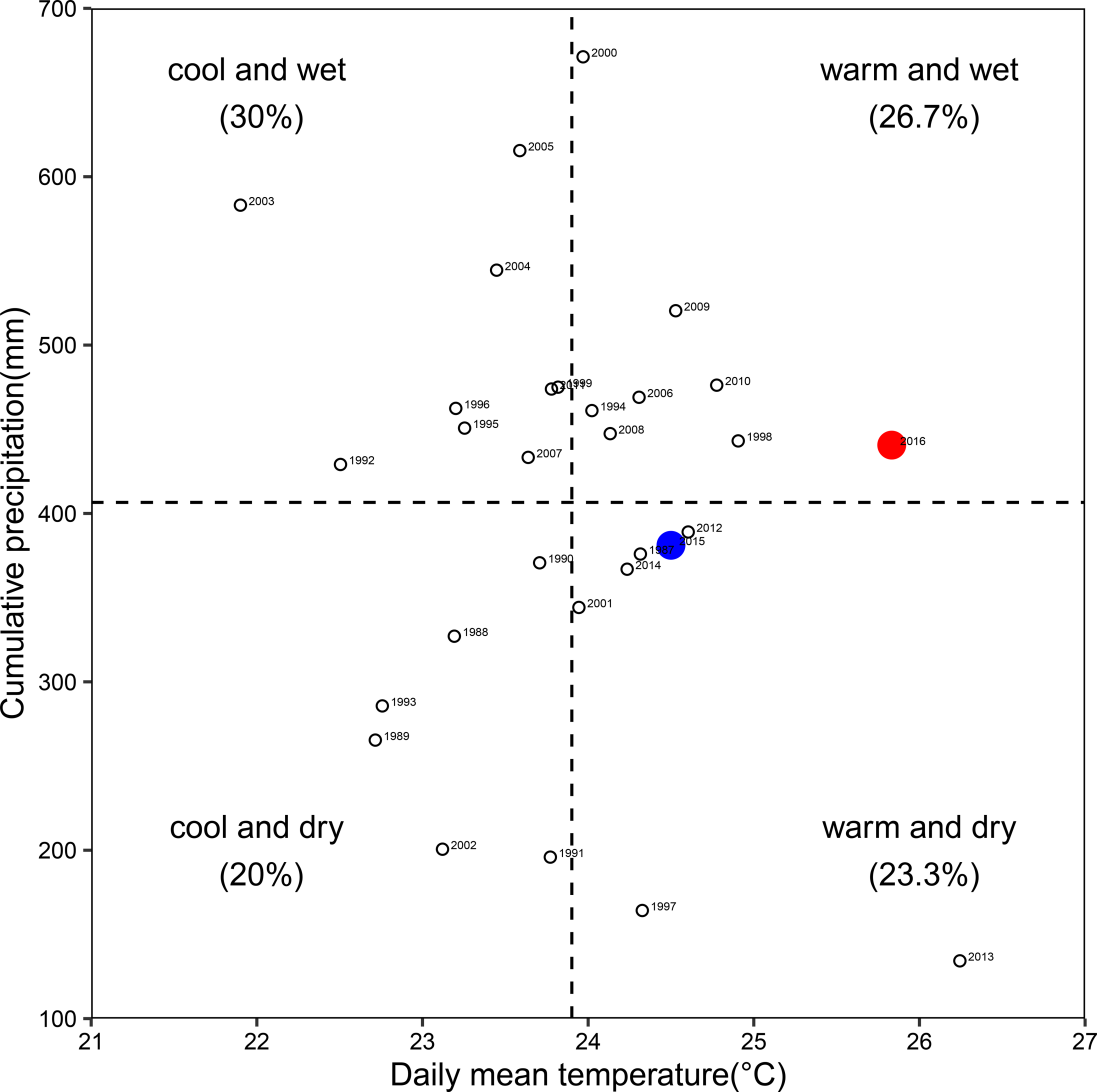
The distribution of daily mean temperature and cumulative precipitation during the corn growing season from April to October, 1987–2016. Data points are labeled with years from 1987 to 2016.

**Figure 3:**
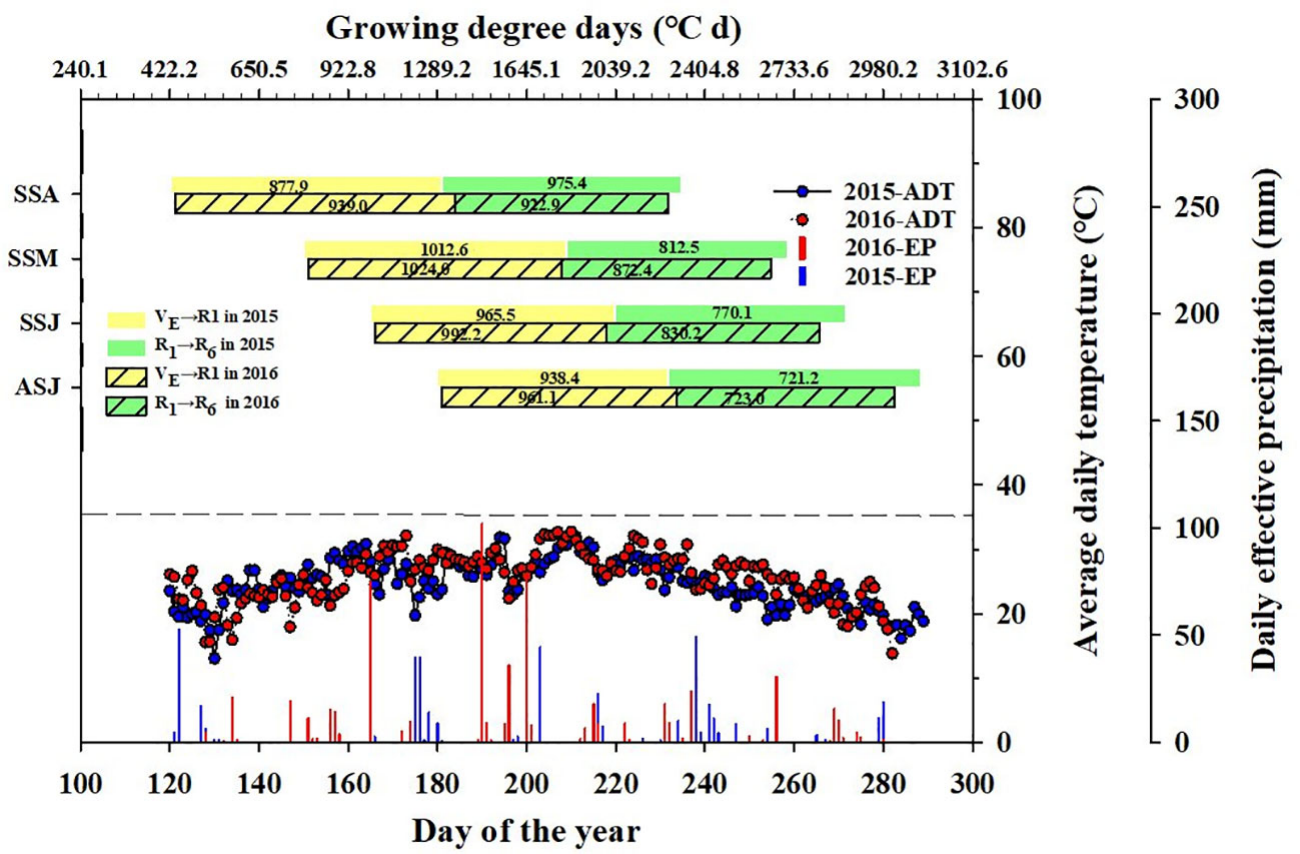
Growing-degree days, average daily temperature and daily effective precipitation during corn growing season for eight sowing dates in 2015 and 2016. VE-R1, vegetative stage; and R1-R6, reproductive stage. Bars are divided by year and growth stages, VE-R1 and R1-R6, for 2015 and 2016, color-coded in yellow and green. Line graph below shows average daily temperature and effective precipitation for 2015 and 2016, with blue and red dots for average daily temperature and vertical lines for daily effective precipitation over the year.

Long-term (1987-2016) weather data such as daily weather data, including the average daily temperature (ADT), daily maximum temperature (*T_max_*), daily minimum temperature (*T_min_*), daily sunshine duration (DSD), and effective precipitation (EP) were obtained from the China Meteorological Data Sharing Service System (http://data.cma.cn/site/article/id/347.html) Zhengzhou station. Daily sunshine hours were the actual duration of sunlight throughout the entire day (Hu et al., 2019; Liu et al., 2010). During the study period, weather data such as temperature and precipitation were collected from an on-site research farm weather station using an HL-10 automatic weather station (Jauntering International Corporation, Taiwan), which was located approximately 500 meters away from the experimental field. The long-term (1987-2016) mean annual temperature and effective precipitation during corn growing season were 23.9°C and 406.6 mm, respectively (**Figure 2**). Compared to the 30-year average, 2016 was considered a hot and wet year and 2015 was close to a normal year. The distribution of growing degree-days, average daily temperature and precipitation during corn growing season in 2015 and 2016 are shown in **Figure 3**. The first killing frost occurred on the October 24^th^, 2015; and October 23^th^, 2016.

The growth and development of crops occur within a range of temperatures, defined by a base temperature (*T_base_*) and an upper temperature threshold (*T_ut_*). In most field environments, temperatures fall between *T_base_* and *T_ut_*, and there is a linear relationship between cumulative temperature and the growth rate. This concept, is known as “GDD” or “heat units”, is used to describe and predict crop development. GDD is a more reliable predictor of crops are development than calendar days because it is not affected by the temperature range in which the crop is grown, provided temperatures remain between *T_base_* and *T_ut_*. Base temperatures vary among crops (Qian et al., 2010). For warm season crop corn, we set *T_base_* and *T_ut_* to 10°C and 35°C, respectively. The GDD was calculated as the following (Cross and Zuber, 1972; McMaster and Wilhelm, 1997):


$$\text{GDD}=\sum_{\text{t}=1}^{N}\left[\left(\frac{T_{\max}+T_{\min}}{2}\right)]-T_{base}\right]$$

Where N, *T_max_*, *T_min_*, and *T_base_* are number of days from sowing to harvesting, the daily maximum temperature, the daily minimum temperature, and the base temperature, respectively. If *T_min_*_<_ *T_base_*, then *T_min_*_=_ *T_base_*. Similarly, if *T_max_* > *T_ut_*, then *T_max_*_=_ *T_ut_*.

### 2.4 Grain filling, grain yield, and yield components

Before silking, the selected corn ears were marked and bagged until the silks grew 2-3 cm long. Then, manual pollination was conducted from 9:00 am to 10:00 am after removing the bags to ensure uniform pollination timing. Silking date, which is defined as the date when at least 50% of plants in a plot have one or more silks emerged from the upper ear, was recorded based on daily observations. To estimate the grain filling rate, kernels were measured from pre-tagged ears in the central rows of each plot. Starting from 10 days after silking, ears from three plants in each plot were sampled at five-day intervals until physiological maturity, indicated by a black layer in the kernel. Kernels were dried at 80°C to a constant weight and weighed. The process of grain filling was fitted with the logistic equation (Richards, 1959), and the grain filling parameters of corn were calculated as follows:


$$W=\frac{\text{A}}{1+{\text{Be}}^{-\text{kt}}}$$

$$G=\frac{{\text{AKB}}^{-\text{Kt}}}{{\left(1+{\text{Be}}^{-\text{kt}}\right)}^{2}}$$

$$G_{\max}=\frac{\text{AK}}{4}$$

$$G_{mean}=\frac{\text{AK}}{6}$$

$$D=\frac{6}{\text{K}}$$

A is the maximum kernel weight (g), *t* is the days after pollination (d), B is the coefficient at the initial stage, and K is the slope of the logistic curve. *G_max_* and *G_mean_* are the maximum and mean kernel growth rates during the effective grain filling period, respectively. *D* is the effective grain filling duration.

At the physiological maturity stage, an area of 2.4 m^2^ from the center of each plot (two central 2-m long rows) was manually harvested, and the weight of the grains was measured. A portable moisture meter (PM8188-A, Kett Electric Laboratory, Tokyo, Japan) was used to determine the grain moisture content. Grain yield was adjusted to a moisture content of 14% and reported. To determine 1000-kernel weight, a random sample of 1000 kernels was selected and weighted.

### 2.5 Statistical analysis

Data of grain yield (GY), kernel number per plant (KNP), and 1000-kernel weight (TKW) were analyzed using the Proc Mixed Model of SAS (Littell et al., 1998), considering sowing date a fixed factor, block a random factor. Since a preliminary data analysis showed a significant interaction between year and sowing date, data were analyzed separately each year. When the sowing date effect was significant at the 5% level, the means comparisons (Least Significant Difference) were performed and the least squares means (e.g., lsmeans) were reported. The grain filling process in the field was simulated using CurveExpert Professional 2.6.3 software (https://www.curveexpert.net/).

Structural equation modelling (SEM) (Schermelleh-Engel et al., 2003) was performed using IBM SPSS Amos version 19 statistical software (Arbuckle, 2010). SEM was chosen to explore the direct and indirect effects of weather factors and grain filling parameters on kernel weight. The overall fit of the model was evaluated using several indicators, including the χ^2^ statistic with the associated probability, the root mean square error of approximation (RMSEA) with the associated probability, and the Bentler-Bonett Index or Comparative Fit Index (CFI). A RMSEA *p*-value greater than 0.05 and a CFI value greater than 0.90 are considered to indicate a good fit of the model. The significance of each path in the model was determined by the probability level (*P*< 0.05).

## 3 Results

### 3.1 Weather during corn reproductive growth stage

**Figure 4:**
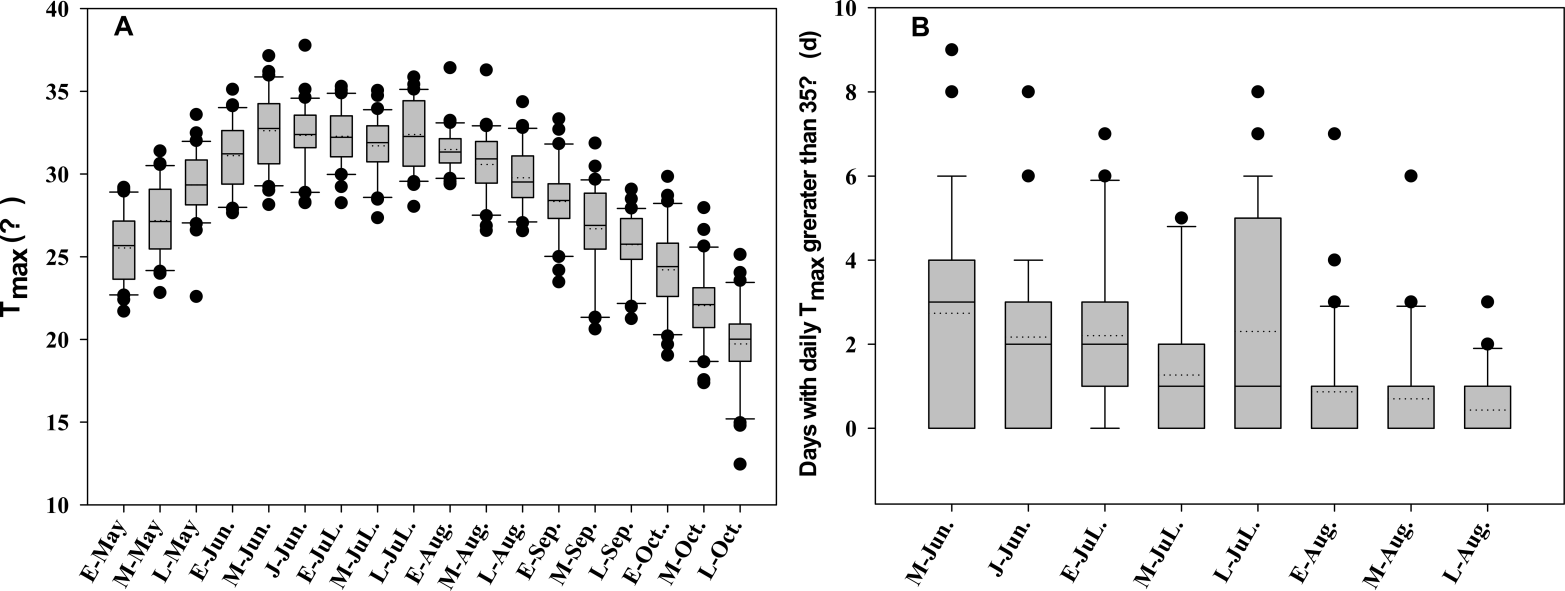
Distribution of **(A)** maximum temperature during corn growing seasons and **(B)** days with T_max_ greater than 35°C during silking stage, 1987 to 2016. E-Apr., M-Apr. and L-Apr. denotes early, middle and late April, respectively, and so on. Solid and dotted lines in the interquartile box plots are the medium and mean, respectively.

Long term (30-year) temperature data showed that peak temperatures during corn growing seasons typically occurs from mid-June to late July (**Figure 4**). Analysis of the number of high-temperature days (>35°C) during the silking stage indicated the likelihood of high temperature declines after early August. The silking stage of corn planted in late April (e.g., SSA) occurred in early June, while the silking stage of early summer corn planted in late May (SSM) fell in early August.

The grain filling window varied due to different sowing dates, ranging from June 20 to Oct 20 in our study. In comparison with the 30-year average (1987-2016), the average daily temperature increased by 2.1% and 7.9% in 2015 and 2016, respectively (**Table 1**). Growing degree-days increased by 3.3% and 12.3% in 2015 and 2016, respectively. Daily sunshine duration decreased by 4.8% in 2015 and increased by 3.2% in 2016, while effective precipitation increased by 8.4% in 2016 and decreased by 6.3% in 2015. Temperature conditions in 2015 was similar to the 30-year average, while those in 2016 were characterized by high temperatures.

**Figure 5:**
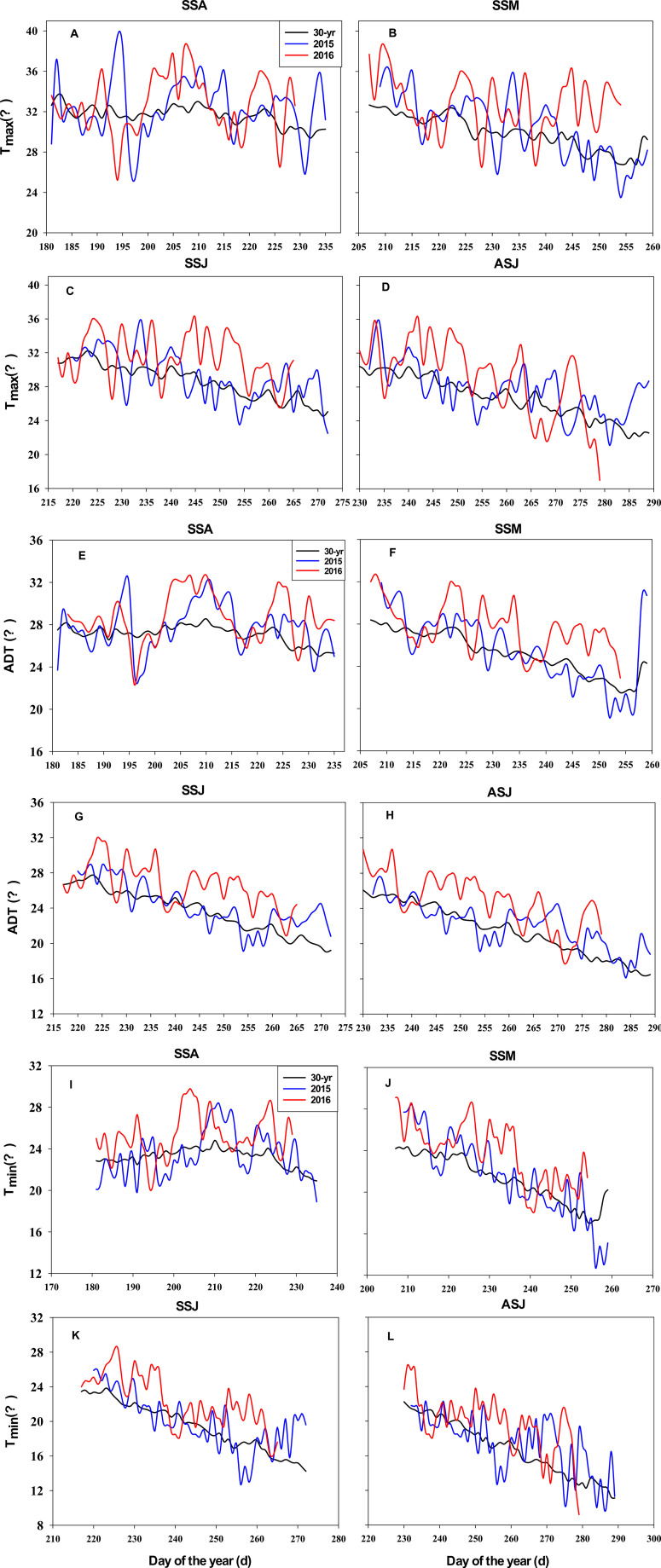
T_max_, **(A-D)** ADT **(E-H)**, and T**_min_ (I-L)** during the corn grain filling period in 2015, 2016 and the 30-yr long-term period (1987-2016). T_max_, average daily maximum temperature; ADT, average daily temperature; T_min_, average daily minimum temperature; SSA, SSM, SSJ and ASJ represent spring sowing in late April, summer sowing in late May, summer sowing in mid-June and autumn sowing in early July, respectively.

There were remarkable variations in GDD, T_max_, and EP during grain filling across eight environments (four sowing dates and two years), and the coefficient of variation was 12.1%, 11.5% and 42.0%, respectively (**Table 1** and **Figure 5**). GDD and ADT averaged across the four sowing dates were 6.7% and 7.1% higher in 2016 than in 2015, respectively. This difference was most evident for the SSM and ASJ sowing dates. The effective precipitation average across the four sowing dates was 14.7% higher in 2016 than in 2015, with the greatest difference being observed for the SSA sowing date, which was 116.4% higher in 2016 than in 2015. Throughout the corn grain filling stage, the SSJ experienced more days of high temperatures, particularly during the early to middle stages of grain filling compared to other sowing dates (**Figure 5**).

### 3.2 Grain yield, kernel number per plant and 1000-kernel weight

**Figure 6:**
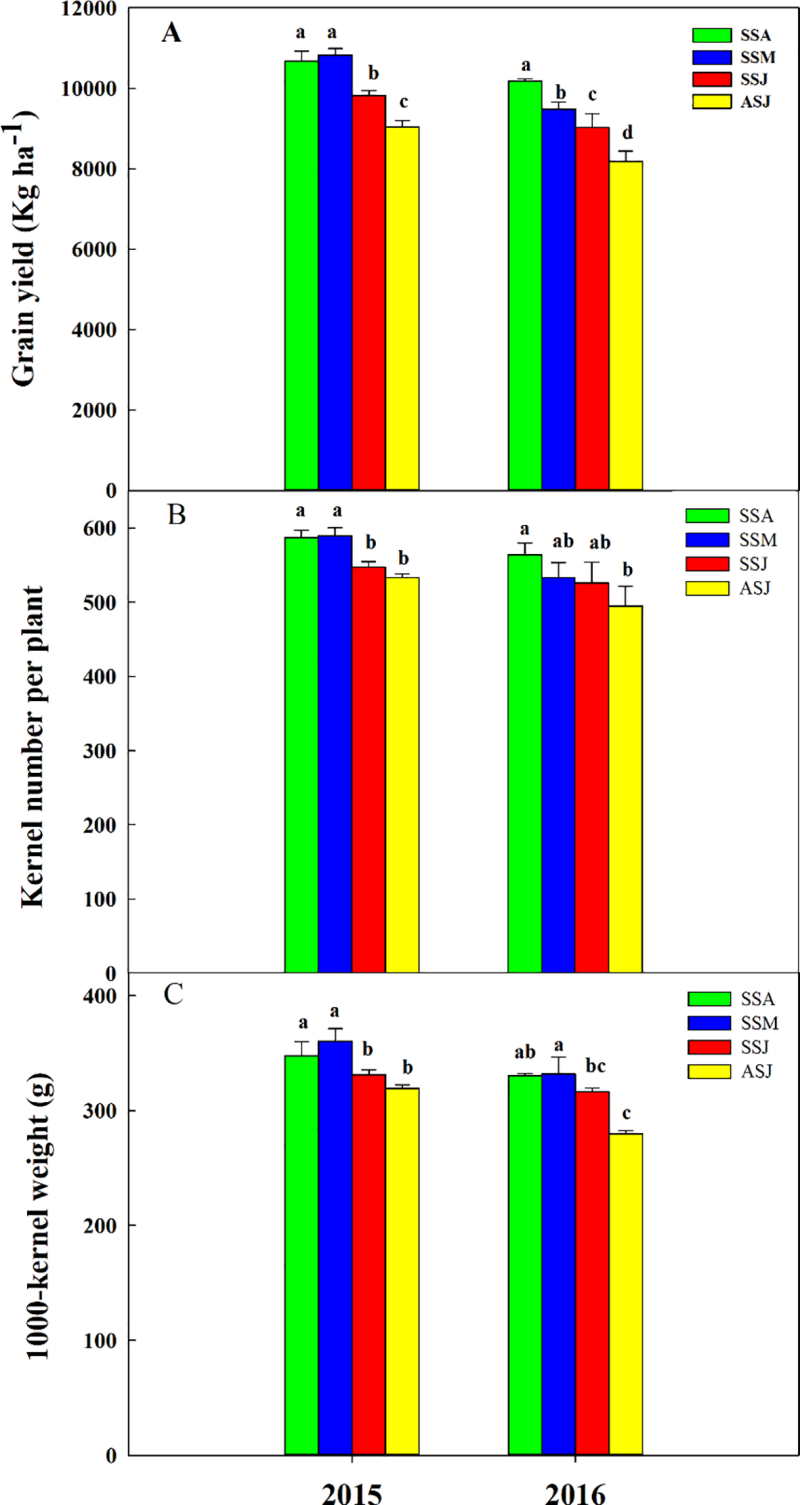
Effects of sowing dates on **(A)** grain yield, **(B)** kernel number per plant, and **(C)** 1000-kernel weight in 2015 and 2016. Bars marked with different letters within a year indicate a significant difference between different sowing dates at the 0.05 probability level. SSA, SSM, SSJ and ASJ were spring sowing in late April, summer sowing in late May, summer sowing in mid-June and autumn sowing in early July, respectively.

In each year, grain yield (*P*< 0.01), kernel number per plant (*P*< 0.01) and 1000-kernel weight (*P*< 0.01) were affected by sowing dates (**Figure 6**). In 2015, GY, KNP and TKW first increased and then decreased as sowing was delayed; while in 2016, GY, KNP, and TKW decreased gradually as a result of delayed sowing. In comparison with the ASJ, the GY increased by 21.1% in SSA, 17.9% in SSM and 9.4% in SSJ, respectively; the KNP increased by 12.0% in SSA, 9.2% in SSM and 4.4% in SSJ, respectively; the TKW increased by 13.3% in SSA, 15.6% in SSM and 8.1% in SSJ, respectively. Therefore, the earlier sowing, the higher GY and the higher KNP and TKW (**Figure 6**).

The differences in GY for various sowing dates ranged from 1.4% to 19.8% in 2015, while the differences ranged from 5.1% to 24.4% in 2016. The average GY, KNP, and TKW across all sowing dates were 8.7%, 6.1%, and 7.4% lower in 2016 than in 2015. The SSM in 2016 encountered high temperatures, with the average daily maximum temperature reaching as high as 36.8°C on the 5th day after tasseling, which was 6.4% higher than the highest temperature recorded during corn tasseling period in 2015. As a result, GY and KNP were 12.4% and 9.5% lower in 2016 than in 2015, respectively.

The GY for the spring-, summer-, and autumn-sown corn were 4.9%, 11.6%, and 10.5% higher in 2015 than in 2016, respectively. KNP for the spring-, summer-, and autumn-sown corn were 4.0%, 7.3%, and 7.7% higher in 2015 than in 2016, respectively. The TKW for the spring-, summer-, and autumn-sown corn were 5.2%, 6.7%, and 14.2% higher in 2015 than in 2016, respectively.

### 3.3 Grain filing of corn kernels

The grain filling parameters derived from the logistic model showed significant differences among different sowing date treatments (**Table 2**). On average, across the four sowing dates, the grain filling duration in a normal year 2015 was 2.97 days longer than in a high temperature year 2016, while the mean grain filling rate was 0.07% higher in 2015 than in 2016. Over the two-year study period, spring-sown corn had a longer active grain filling period, with a 1.40-day increase compared to summer-sown corn and a 3.47-day increase compared to autumn-sown corn. In contrast, summer-sown corn showed a 0.8% increase in mean grain filling rate compared to spring-sown corn, and a 5.6% increase compared to autumn-sown corn.

**Table 2:** Estimated parameters for the grain filling logistic model W = $\;\frac{\text{A}}{1+{\text{Be}}^{\text{-kt}}}$ of corn seeded on different dates, 2015 and 2016.

Year	Treatments	A	B	K	*G*_max_	*G_mean_*	*D*
2015	SSA	34.33b	29.19c	0.140c	1.21b	0.81b	42.77a
	SSM	35.92a	32.28bc	0.143c	1.28a	0.85a	42.11a
	SSJ	32.96c	33.31b	0.149b	1.23b	0.82b	40.18c
	ASJ	31.80d	42.53a	0.156a	1.23b	0.82b	38.60c
2016	SSA	33.75a	38.98b	0.153c	1.29a	0.86a	39.23a
	SSM	32.60b	39.52b	0.157b	1.28a	0.85a	38.19b
	SSJ	31.31c	38.39b	0.158b	1.24b	0.83b	37.92c
	ASJ	27.82d	55.11a	0.165a	1.14c	0.76c	36.46c

A is the maximum kernel weight (g), B is the coefficient at the initial stage and K is the slope of the logistic curve. *G_max_*, maximum grain filling rate (g 100 grain^-1^d^-1^); *G_mean_*, grain filling rate (g 100 grain^-1^d^-1^); *D*, grain filling duration (days). Parameters within a column marked with different letters within a year indicate a significant difference at the 0.05 probability level. SSA, SSM SSJ and ASJ represent sowing dates in late April, late May, mid-June and early July, respectively.

**Figure 7:**
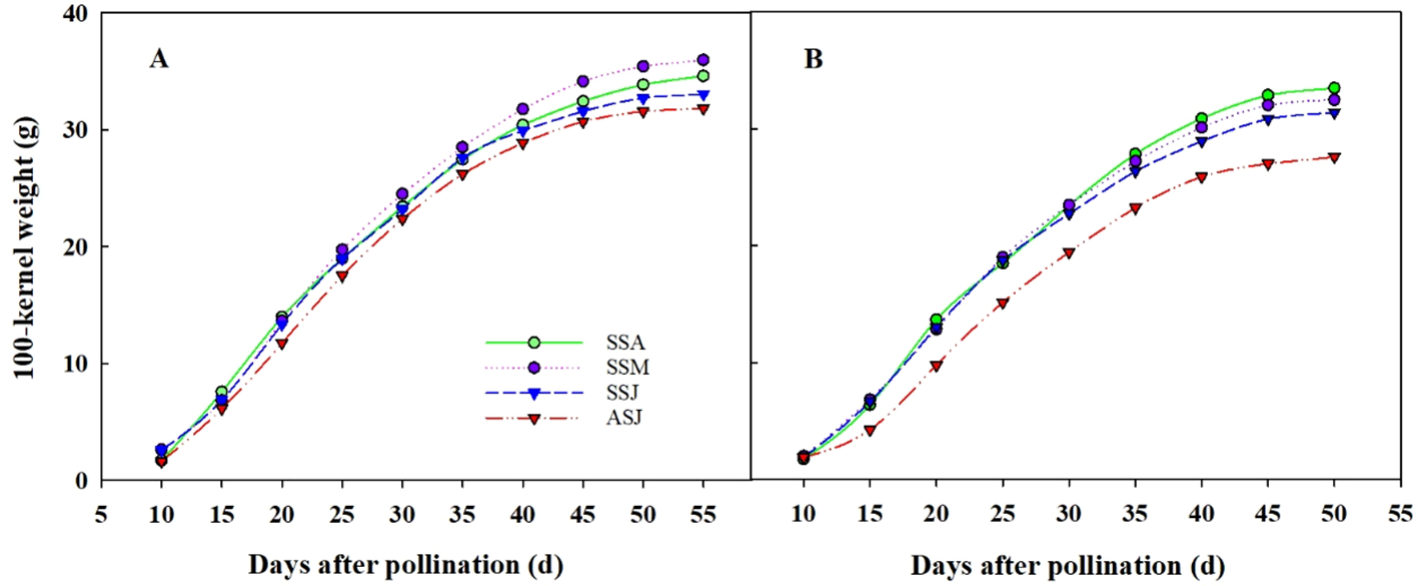
The dynamic change of grain filling of corn sown on different sowing dates, **(A)** 2015 and **(B)** 2016. SSA, SSM, SSJ and ASJ were spring sowing in late April, summer sowing in late May, summer sowing in mid-June, and autumn sowing in early July, respectively.

The grain filling process, measured by the change of kernel weight (KW) over days after pollination followed a logistic model pattern (**Figure 7**). Significant differences in KW were observed among sowing dates during the early grain filling stage, and the mean grain filling rate was 14.2% lower in 2016 than in 2015 (**Figure 7** and **Table 3**). Significant differences in KW were observed among sowing dates during the middle grain filling stage, with the weight gain following the order: ASJ< SSJ< SSA< SSM. In 2015, the mean grain filling rate was 7.4%, 4.0%, and 1.2% higher for SSM than for SSA, SSJ, and ASJ, respectively. In 2016, the mean grain filling rate was 0.8%, 4.2%, and 12.7% higher for SSA than for SSM, SSJ, and ASJ, respectively. During the late grain filling stage, although the KW for all treatments gradually increased, the value for SSM was the largest and significantly higher than other sowing dates in 2015. KW was 4.0%, 8.1%, and 11.5% higher for SSM than for SSA, SSJ, and ASJ, respectively. The highest KW was achieved for SSA in 2016, with KW being 3.1%, 6.6%, and 27.6% higher for SSA than for SSM, SSJ, and ASJ, respectively.

**Table 3:** Effects of sowing dates on grain filling traits during early, middle and late grain filling stages of corn, 2015 and 2016.

Year	Treatments	Early grain filling stage		Middle grain filling stage		Late grain filling stage	
		Days(d)	MGR (g 100 grains^-1^ d-^1^)	Days(d)	MGR (g 100 grains^-1^ d-^1^)	Days(d)	MGR (g 100 grains^-1^ d-^1^)
2015	SSA	15.1b	0.482b	19.1a	1.045c	23.7a	0.293c
	SSM	15.1b	0.502a	18.5b	1.122a	23.0b	0.314a
	SSJ	14.7c	0.475b	17.6c	1.079b	22.0c	0.302b
	ASJ	16.0a	0.419c	16.5d	1.108a	20.6d	0.311a
2016	SSA	15.0b	0.465a	17.2a	1.132a	21.4a	0.317a
	SSM	15.3c	0.459b	16.8b	1.123a	20.9b	0.315a
	SSJ	14.7d	0.449c	16.7b	1.086b	20.7b	0.304b
	ASJ	16.4a	0.359d	16.0c	1.004c	19.9c	0.281c

The estimated parameters within a column for each year marked with different letters indicate a significant difference at the 0.05 probability level. MGR, mean grain filling rate; SSA, SSM, SSJ and ASJ represent sowing dates in late April, late May, mid-June and early July, respectively.

### 3.4 Relationships between weather factors and grain filling

**Figure 8:**
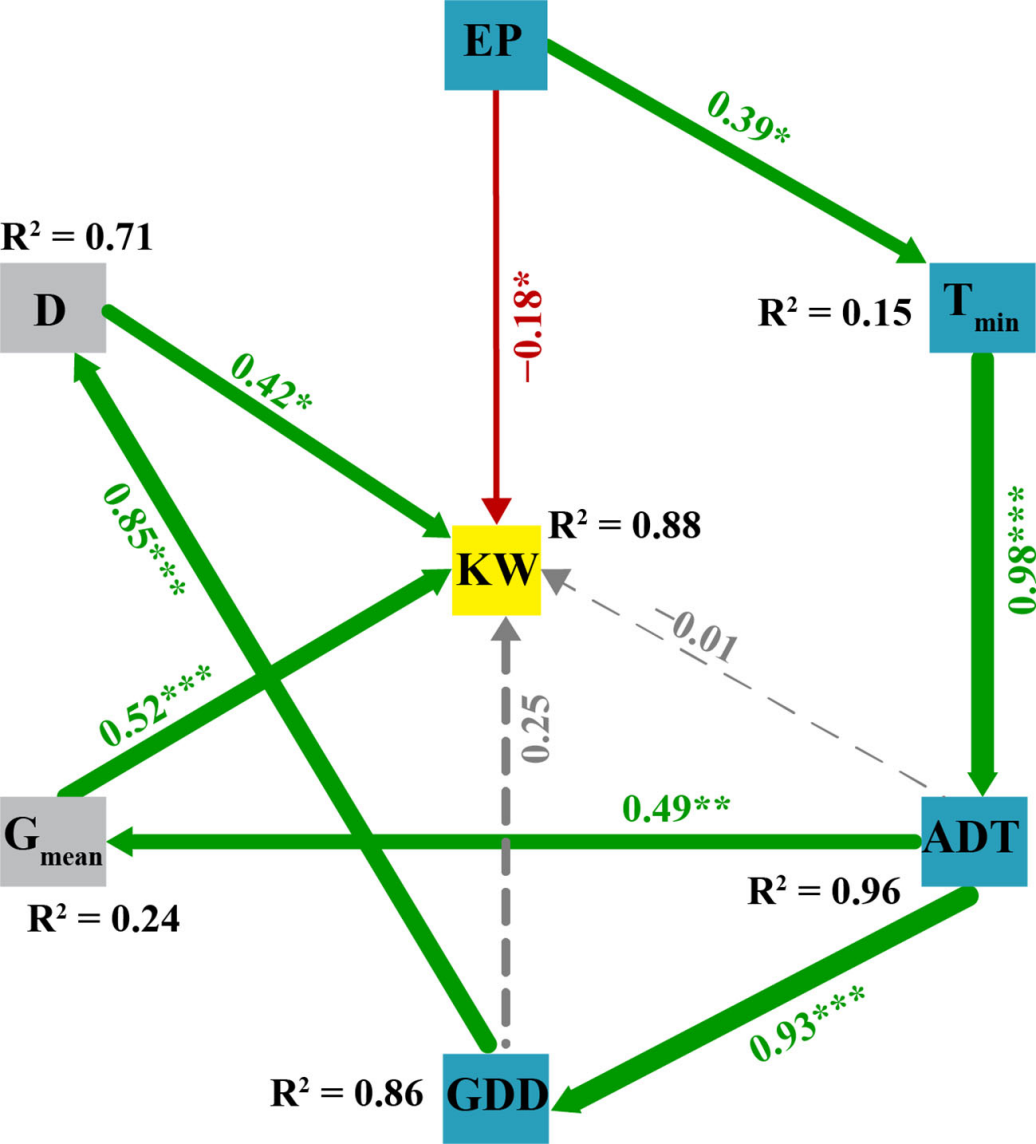
The structural equation model (SEM) illustrating the direct and indirect effects of weather factors, grain filling parameters and KW. Variables inside the boxes are measured variables entered in the model. Numbers adjacent to arrows are standardized path coefficients, analogous to partial regression weights and indicative of the effect size of the relationship. ***p< 0.001; **p< 0.01; *p< 0.05. Continuous and dashed arrows indicate positive and negative relationships, respectively. The path widths are scaled proportionally to the path coefficients. As in linear models, *R^2^* indicates the proportion of variance explained. SEM showed a good fit to the data, as indicated by criteria of CFI (0.91) and RMSEA (*P* = 0.28). KW, kernel weight; *G_mean_*, mean grain filling rate; *D*, active grain filling day; GDD, growing degree-days; ADT, average daily temperature; T_min_, average daily minimum temperature; and EP, effective precipitation.

A structural equation modeling analysis was conducted to examine the relationship between weather factors and grain filling parameters. It was found that specific weather factors had a significant impact on grain filling parameters (**Figure 8**). SEM explained 88% of variation in kernel weight, 96% in average daily temperature, 86% in growing degree-days, 71% in grain filling duration and 24% in mean grain filling rate. The results showed that the grain filling duration and rates had direct positive effects on kernel weight with path coefficients of 0.42 and 0.52, respectively. However, effective precipitation had direct negative effects on kernel weight with a path coefficient of -0.18. The growing degree-days were strongly associated with the grain filling duration (path coefficient: 0.85) and indirectly affected the kernel weight through the grain filling duration with the product of path coefficients of 0.36. Furthermore, the average daily temperature was strongly associated with the mean grain filling rate (path coefficient: 0.49) and had indirect positive effects on kernel weight (product of path coefficient of 0.25), primarily through the grain filling rate.

## 4 Discussion

### 4.1 High temperature during grain filling reduced grain yield

High temperatures are predicted to become more intense and more frequent, potentially creating

disadvantageous conditions during the reproductive growth stage for crops such as corn, wheat, soybean, and rice, leading to a significant reduction in grain yield (Eyshi Rezaei et al., 2015; Gabaldón-Leal et al., 2016; Lobell et al., 2013; Mastilović et al., 2017; Siebers et al., 2015). Historical climate change trend from 1961 to 2015 resulted in a simulated yield decrease of 161.2 kg ha^-1^ per decade, mostly due to a significant temperature increase of 0.43°C per decade (Zhang et al., 2019). Grain yield was highly correlated with kernel number and kernel weight (Mayer et al., 2014; An et al., 2018; Villegas et al., 2015). The average 1000-kernel weight and kernel number per plant across the four sowing dates was 8.0% and 6.5% higher in 2015 than in 2016, respectively, due to the high temperatures during grain filling in 2016. Mayer et al. also reported a similar observation that kernel weight experienced a rapid decline during the grain filling period under high temperature (Mayer et al., 2014). An early termination of grain filling under high temperature was not attributed to early leaf senescence resulting from the lack of assimilate, but rather to the abortion of kernels, resulting in reduced sink activity (Mayer et al., 2014). Consequently, the average grain yield was 9.5% higher in 2015 than in 2016 due to high temperature ripening.

The weather changes over the past forty-five years at the experimental site was consistent with findings from previous studies conducted in other regions of the NCP (Tao et al., 2016a; Wang et al., 2012; Xiao and Tao, 2016; Zhang et al., 2015). Our study demonstrated that the weather change trend during the corn growth period included increased temperature, decreased cumulative sunshine durations, and reduced effective precipitation (**Table 1**). The historical average corn yield was 5249.9 kg ha^-1^ in the NCP from 1981 to 2009 (Tao et al., 2016a). Compared with this 29-year average, the

corn yield increased by 92.2% in 2015 and by 75.5% in 2016. These yield increases may be attributed to the development of corn cultivars with greater stress tolerance (Ci et al., 2011; Gouache et al., 2012) and improvements of agronomic management practices (Chen et al., 2013), such as alteration of sowing dates. Adjusting sowing dates proved to be an effective strategy for examining the influence of various weather factors on crop yield (Tsimba et al., 2013). To mitigate the impact of weather variation on corn production, optimizing sowing date is critical to maximize the utilization of temperature, thermal and water resources, thus improving yields.

### 4.2 Early sowing achieved high yield

Studies showed that the prolonged heat stress during corn silking reduced kernel number per plant (KNP) (Ordóñez et al., 2015; Wang et al., 2019). Specifically, Wang et al. (2019) found that exposure to high temperature of 40°C significantly reduced KNP. Another study reported that corn kernels exposed to 35°C temperature stress for either 4 or 8 days showed different abortion rates: 97% for prolonged (8 days) stress and about 23% for short-term (4 days) stress (Cheikh and Jones, 1994). This suggests that extended high temperatures during silking lead to kernel abortion, whereas shorter heat stress periods have minimal impact on KNP. In our study, we observed a 3.5% decrease in KNP in SSM than in SSA in 2016. During the silking stage of SSM in 2016, effective precipitation and cumulative sunshine duration were recorded at 52.4 mm and 41.3 hours, respectively. The combined stress of waterlogging and shading during the silking stage inhibited the growth of ovaries and silks at the tip ear, resulting in a reduction in the number of emerged silks and total kernel number per plant (Zhou et al., 2021; Ren et al., 2023).

Temperature was a critical factor that influenced post-silking dry matter accumulation and grain yield (Zhou et al., 2016). Previous studies suggested that longer grain filling duration increase yield by promoting kernel dry matter accumulation (Liu et al., 2010; Zhu et al., 2018). The average active grain filling duration across the four sowing dates was 7.2% longer in 2015 than in 2016, while the mean grain filling rate was 0.7% higher in 2016 than in 2015. Our findings showed that the high temperatures during grain filling periods shortened the effective grain filling periods while increasing mean grain filling rates. However, the increased grain filling rate was insufficient to compensate for the yield losses caused by the shortened grain filling duration. As a result, high temperatures during grain filling shortened the active grain filling period and greatly reduced kernel weight and grain yield.

Given the harmful effects of high temperatures on crop yields, researchers suggested various adaptation strategies to mitigate the reduction in corn production. These strategies include adjusting sowing dates, selecting appropriate cultivars, and irrigation (Lv et al., 2020; Tack et al., 2017), while research showed that adjusting sowing date did not mitigate the heat stress around silking (Gao et al., 2021). Sowing date had varying effects on the weather conditions during the growth period, including changes in solar radiation and temperature (Ji et al., 2024), which can affect the process of grain filling of corn (Yan et al., 2017). Altering sowing dates may be an appropriate agronomic measure to avoid creating an unfavourable environment for crops (Opsi et al., 2013) and better adapt to changing climate conditions (Bonelli et al., 2016; Iizumi et al., 2014). Early sowing tended to minimize terminal high temperature (Paudel et al., 2023). Studies showed that delaying the sowing date led to a 1% decrease in grain yield per day, which was attributed to low temperatures during crop vegetative growth, elevated temperatures during the grain filling period, and a short duration of grain filling (Shah et al., 2020).

Our results indicated that growing degree-days had weak direct positive, but strong indirect influence on kernel weight through the grain filling duration. The early summer-sown corn increased growing degree-days by 11.4%, compared with the summer-sown corn. Early sowing could increase growing degree days and promote indirect effects on kernel weight through prolonging the active grain filling duration. The contribution of grain filling stage to the final kernel weight was in the following order: middle (57.7%), early (21.1%) and late (20.1%) stages. In 2016, the early summer-sown corn exhibited a 4.1% increase in early grain filling duration, a 3.4% increase in grain filling rate for the middle grain filling duration and a 3.6% increase in grain filling rate for the late grain filling duration compared to the summer-sown corn. Thus, the early summer-sown corn increased kernel weight by prolonging the grain filling duration, especially during the early grain filling stage, and increasing grain filling rate for the middle and late grain filling durations.

### 4.3 Average daily temperature and growing degree days were key drivers of kernel weight

SEM was used as an effective method to identify both direct and indirect relationships among various variables (Grace, 2006). In the current study, the grain filling duration and mean grain filling rates had direct and positive effects on kernel weight, which is consistent with previous research suggesting that variations in final kernel weight were associated with changes in kernel growth rate and the active grain filling period (Hatfield, 2014). Research showed that precipitation and temperature are major limiting factors of crop production in Inner Mongolia (Li et al., 2022). In our study, effective precipitation had direct and significant negative effects on kernel weight, likely due to waterlogging and shading during silking, which results in low assimilation availability, poor pollination, and reduced kernel number per ear (Gao et al., 2018). As corn was irrigated and no water stress was observed during growing season, excessive precipitation could potentially reduce grain yield. On the other hand, the impact of sunshine duration on kernel weight was minimal, as it could meet the demands of the late grain filling stage and was not a limiting factor for the final kernel weight (Hu et al., 2019).

A previous study showed that effective grain filling period was correlated with growing degree-days, and kernel growth rate was influenced by the average daily temperature, with kernel weight strongly correlated with growing degree-days (Zhou et al., 2017). In our study, growing degree-days had weak and direct positive effects on kernel weight (path coefficient>0.1, but *P*>0.5), while it had strong indirect effects on kernel weight through the grain filling duration. In addition, the average daily temperature had weak direct negative effects but a significant indirect positive influence on kernel weight through mean grain filling rate. The findings indicated that planting corn earlier could increase growing degree-days and higher average daily temperature, thereby strengthening the indirect effects on kernel weight through the grain filling duration and mean grain filling rate.

## 5 Conclusion

In conclusion, this two-year study demonstrated that weather conditions during the grain filling period had a significant impact on the yield and grain filling process of corn in the North China Plain region. High temperatures during the grain filling period had a negative impact on the yield, reducing kernel weight and active grain filling duration. Compared with the normal temperatures in 2015, the active grain filling duration has decreased by 7.2%, and the grain yield has decreased by 8.7% in 2016. The results also showed that the increased mean grain filling rate was insufficient to compensate for the yield loss caused by the reduction in active grain filling duration. The findings suggested that advancing the sowing date of summer corn from mid-June to late May prolong the active grain filling duration, offering a potential management practice to mitigate high temperature-induced yield loss. This study provides valuable insights for optimizing sowing dates to address the impacts of anticipated high temperatures resulting from climate change on corn production in the North China Plain region.

## Data Availability

The raw data supporting the conclusions of this article will be made available by the authors, without undue reservation.
